# Interaction of drugs of abuse and microRNA with HIV: a brief review

**DOI:** 10.3389/fmicb.2015.00967

**Published:** 2015-09-29

**Authors:** Sudheesh Pilakka-Kanthikeel, Madhavan P. N. Nair

**Affiliations:** Department of Immunology, Institute of Neuroimmune Pharmacology, Herbert Wertheim College of Medicine, Florida International UniversityMiami, FL, USA

**Keywords:** HIV, microRNA, drugs of abuse, cocaine, latency

## Abstract

MicroRNAs (miRNAs), the post-transcriptional regulators of gene expression, play key roles in modulating many cellular processes. The changes in the expression profiles of several specific miRNAs affect the interactions between miRNA and their targets in various illnesses, including addiction, HIV, cancer etc. The presence of anti-HIV-1 microRNAs (which regulate the level of infectivity of HIV-1) have been validated in the cells which are the primary targets of HIV infection. Drugs of abuse impair the intracellular innate anti-HIV mechanism(s) in monocytes, contributing to cell susceptibility to HIV infection. Emerging evidence has implicated miRNAs are differentially expressed in response to chronic morphine treatment. Activation of mu opioid receptors (MOR) by morphine is shown to down regulate the expression of anti-HIV miRNAs. In this review, we summarize the results which demonstrate that several drugs of abuse related miRNAs have roles in the mechanisms that define addiction, and how they interact with HIV.

## Introduction

MicroRNAs (miRNAs) are short non-coding regulatory RNAs, approximately 22 nucleotides in length, which bind to the 3′ untranslated regions (UTR) of messenger RNAs (mRNAs) and interfere with their translation, thus contributing to a significant post-transcriptional regulatory step in gene expression (Bartel, 2004; Pilakka-Kanthikeel et al., 2011). One of the important host innate defense mechanisms against retroviruses, such as HIV, is the presence of intracellular viral restriction factors. The micro RNAs belong to this group of “restriction factors.” Over 3000 mature miRNAs have been identified in various species, which highlight their importance in gene regulation. The effect of miRNA can be either direct or indirect. Host cellular miRNA can target host genes/proteins involved in the HIV replication or target viral genes to post transcriptionally silence the protein production. Similarly, viral miRNA (viRNAs) can also either target viral genes, cellular mRNAs or miRNAs. Since their discovery, miRNAs have been linked to biological processes such as drug addiction (He et al., 2010; Zheng et al., 2010), pain perception (Kusuda et al., 2011), neuron development (Gao, 2015), viral infection (Dave and Khalili, 2010; Wang et al., 2011), and opioid receptor regulation (Wu et al., 2008; Sanchez-Simon et al., 2010). Although the mechanisms not completely understood, evidences support the notion that HIV-1 down-regulates some of the cellular anti-HIV-1 miRNAs by inhibiting the proteins involved in its biogenesis and maturation, as a strategy to persist.

**Figure 1:**
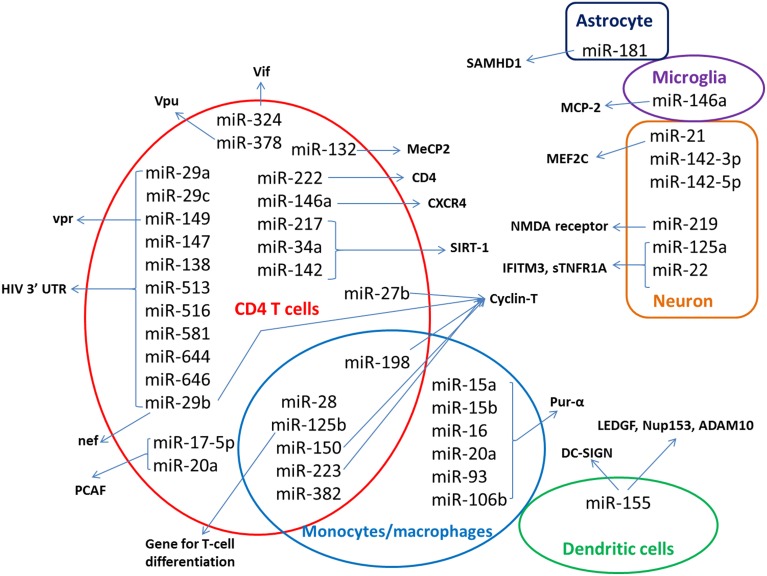
**Host miRNAs implicated in HIV**. Host miRNA that have been identified to have role on HIV replication are shown in the figure, with respect to the cells where they have major role. The host or viral targets of the miRNAs are indicated by the arrows. miRNAs identified from patient samples were not included in the figure.

HIV latency is a stage in which proviral DNA integrated in to the host's genome does not actively replicate. Even though the mechanisms of establishment and maintenance HIV-1 latency are not completely understood, it is believed to be a multifactorial process involving different cellular and molecular mechanisms. miRNA are also reported to be involved in the maintenance of HIV latency (Coiras et al., 2009; Van Lint et al., 2013; Battistini and Sgarbanti, 2014). The miRNA expression profiles seem to be modulated in HIV-1-infected cells and in patients. In this review, we summarize the miRNAs that have been reported to be associated with HIV, their change in expression and how the drugs of abuse modulate their expression. mi-RNAs that have been reported to have role in HIV have been summarized in Figure 1. Functions and other details have been discussed in the following sections.

### Cellular anti-HIV miRNAs in HIV infection: *in vitro* observations

miRNAs have shown to limit HIV-1 infection by multiple mechanisms. They exhibit cell-type-specific expression and differential expression based on cellular differentiation or activation states contributing to differential cellular susceptibility to HIV-1 infection. This feature led the scientists to focus miRNA as “restriction factor” in the recent past. Few studies have identified cellular miRNAs that target a set of accessory genes of HIV-1 or other host genes/proteins involved in the HIV replication. Lecellier et al. reported for the first time the antiviral activity of cellular miRNAs, where they showed significantly enhanced primate foamy virus type 1 (PFV-1) replication by knock down of miR-32 expression (Lecellier et al., 2005). The initial studies opened an exciting avenue of research exploring how to manipulate endogenous miRNAs and alter cellular susceptibility to HIV-1 infection.

#### Anti-HIV miRNAs in CD4^+^T cell

Hariharan et al. using computational approach, predicted five human T-cell miRNAs (miR-29a, miR-29b, miR-149, miR-378 and miR-324-5p) targeting the highly conserved regions across all clades of HIV-1 using computational approach. Among these, miR-29a and miR-29b target the nef gene, whereas miR-149, miR-378, and miR-324-5p target vpr, vif, and vpu respectively (Hariharan et al., 2005). Later Nathans et al. identified 11 miRNAs (miR-29a, 29b, 29c, 149, 147, 138, 513, 516-5p, 581, 644, and 646) that target 3′ UTR of HIV-1 through target prediction analysis of nef, vpr, vif, and vpu (Nathans et al., 2009). They found that the inhibition of miR-29a, b, or c increased HIV-1 production; however, highest effect was associated with miR-29a.

Huang reported five host-coded miRNAs (miR-28, miR-125b, miR-150, miR-223, and miR-382), that target the 3′ UTR of viral mRNAs that inhibit HIV-1 infection (Huang et al., 2007). These anti-HIV miRNAs were differentially expressed in resting vs. active CD4^+^T cells *in-vitro*; significantly higher in resting than in activated CD4^+^T cells. These miRNAs were down-regulated during the activation of resting CD4^+^T cells, correlating with HIV-1 susceptibility. These cellular miRNAs were thought to be contributing to viral latency observed in quiescent cells. The combination treatment of the five anti-HIV-1 miRNAs inhibitors were more effective in substantially increasing the HIV-1 infection compared to the individual inhibitors in resting CD4^+^T cells, but not in activated CD4^+^T cells. These miRNA inhibitors, however, did not affect cellular proliferation status (Huang et al., 2007). miR-125b has been demonstrated to regulate a network of genes in CD4^+^T cells that are critical for its differentiation (Rossi et al., 2011), and responsible for latency induction in naıve CD4^+^T cells (Huang et al., 2007; Wang et al., 2009). Yeung et al. reported a downregulation in miRNA profile (miR-93, miR-148b, miR-221, and miR-16) in HeLa cells after HIV-1 protein expression (Yeung et al., 2005).

miR-27b, miR-29b, miR-150, miR-198, and miR-223 are another group of miRNAs found to be low in resting CD4^+^T cells. They seem to be regulating Cyclin T1 protein (Nathans et al., 2009; Chiang et al., 2012), the expression of which is required for transactivation by HIV-1 Tat. CD4^+^T cell activation resulted in down-regulation of these miRNAs with a subsequent up-regulation of Cyclin T1, which correlated with enhanced HIV-1 susceptibility. However, miR-198 does not undergo downregulation after T cell activation. It is suggested that a cellular negative-feedback loop is triggered during cyclin T1 upregulation resulting in elevated levels of miR-198 and which in turn result in subsequent dampening of the induction of cyclin T1 (Sung and Rice, 2009). miR-27b also acts in a Cyclin T1-dependent manner, overexpression of which decrease viral replication (Chiang et al., 2012).

A study by Chiang et al. reported a relationship between miR-132 and HIV-1 infection. They found significantly lower miR-132 expression in resting CD4^+^T cells than in activated cells. Ectopic expression of miR-132 increased the HIV-1 infection in Jurkat T cells (Chiang et al., 2013), though the exact mechanism behind this effect and the specific step on the viral life cycle that seems to be potentiated by miR-132 are not clear. A probable explanation is that miR-132 overexpression decreases the expression of a cellular protein MeCP2, inhibition of which augments HIV-1 replication (Leoh et al., 2013). However, the precise role of miR-132 and MeCP2 in HIV-1 replication has not been identified. But a study by Desplats et al. reported significantly up-regulated MeCP2 levels in frontal cortex of patients with neurocognitive deficits who had latent HIV-1 infection in the brain (Desplats et al., 2013).

MicroRNAs have also shown to target the receptors and co-receptors needed for HIV entry, thereby restricting the viral entry. Orecchini et al. suggested a mechanism by which Tat manipulate the CD4 receptor, by miR-222 up-regulation (Orecchini et al., 2014). Further studies are warranted in this since the effect of prior induction of miR-222 on viral infection was not investigated by Orecchini et al. Spinello et al. reported a relationship between miR146a and CXCR4 co-receptor. Resting CD4^+^T cells have high expression of miR-146a, which was downregulated during their activation by PHA. High expression of miR-146a inhibits the expression of the co-receptor CXCR4, and prevents the HIV entry in U937 and resting CD4^+^T cells (Spinello et al., 2011; Quaranta et al., 2015).

Tat exposure significantly up-regulates miR-217 and miR-34a, which bind to the 3′ UTR region of SIRT1 mRNA inhibiting its expression. Lower SIRT1 expression is associated with an enhancement in HIV-1 Tat-mediated transactivation (Zhang et al., 2012a,b). Chaudhuri et al. also demonstrated the functional relevance of cellular miRNAs that target SIRT1 in their simian immunodeficiency virus (SIV) encephalitis study. Post-SIV infection, a significant upregulation of miR-142 was noted that leads to down-regulation of SIRT1, potentially contributing to SIV replication and SIV-induced encephalitis (Chaudhuri et al., 2013). Another study showed significant up-regulation of miR-182 expression by Tat. Higher the miR-182 expression, the lower is the expression of nicotinamide phosphoribosyltransferase (NAMPT), which is a regulator of SIRT1. The down-regulation of NAMPT decreases expression of SIRT1 levels, which in turn enhanced HIV-1 Tat transactivation (Chen et al., 2013).

#### Anti-HIV miRNAs in monocyte/macrophage

Monocytes, monocyte derived macrophages (MDM) and monocyte derived dendritic cells (MDDCs) are differentially susceptible to HIV infection. It is suggested that monocyte differentiation and HIV-1 susceptibility are linked by a common set of miRNAs. The five anti-HIV-1 miRNAs (miR-28, miR-125b, miR-150, miR-223, and miR-382), which Huang et al. previously reported, were also highly expressed in monocytes similar to resting CD4 T cells, relating to the refractory nature of monocytes to HIV infection. They showed that level of HIV infectivity was inversely correlated with the level of miRNA expression. These miRNAs were downregulated during the differentiation of monocytes to MDMs (Wang et al., 2009), correlating with the increased susceptibility of macrophages to HIV infection compared to monocytes. Modulating the expression of anti-HIV miRNAs was sufficient to reverse the severity of HIV-1 infection. For e.g., the suppression of these anti-HIV-1 miRNAs in monocytes facilitates HIV-1 infectivity, whereas increase of the antiHIV-1 miRNA expression in macrophages inhibited HIV-1 replication.

However, a subsequent study by Sisk et al. found only miR-223 to be down-regulated during the differentiation of monocytes to MDMs. According to them, the rest of the miRNAs were either higher in MDMs or remain unchanged compared to monocytes (Sisk et al., 2012). The reason for this contradiction is not clear. The different platforms for studying miRNAs, different experimental protocols for the monocyte isolation and/or the monocyte-to-macrophage differentiation, possible alterations in abundance of macrophage subpopulations etc., may have contributed to this difference. Recently, a study by Mestdagh P et al. evaluated the different miRNA quantification platform (Mestdagh et al., 2014).

miR-198 was significantly downregulated upon differentiation of monocytes to macrophages, unlike during T cell activation (Sung and Rice, 2009). miR-198 is shown to be capable of downregulating cycin-T1 protein expression without affecting cyclin T1 mRNA levels. Low miR-198 expression during macrophage differentiation in turn results in increased Cyclin T1 expression and enhances HIV-1 replication within macrophages (Liou et al., 2002).

#### Anti-HIV miRNAs on immune cell differentiation

In addition to their direct effect on HIV-1 replication, miRNAs also plays significant roles in host innate immune defense regulation. Monocyte differentiation into MDDC is regulated and coordinated by different miRNAs (Wang et al., 2009; Noorbakhsh et al., 2010). miR-146a and miR-155 are two important miRNAs involved in innate immunity by regulating the acute immune response following toll like receptor (TLR) stimulation.

TLR3 and TLR4 ligand [poly(I:C) and LPS] stimulation increase miR-155 in the human monocytic cell line THP1, primary human macrophages (Swaminathan et al., 2012) and in murine bone-marrow-derived macrophages (Taganov et al., 2006; O'Connell et al., 2007). miR-155 participates in the maturation of human dendritic cells (DC) and dendritic cell-specific intercellular adhesion molecule-3-grabbing non-integrin (DC-SIGN) expression through the transcription factor PU.1 (Martinez-Nunez et al., 2009). DC-SIGN, which binds to the HIV-1 envelope glycoprotein gp120 is important in the process of trans-infection (Geijtenbeek et al., 2000). Since miR-155 decreases DC-SIGN expression, it has been suggested that miR-155 could prevent entry of HIV through DC-SIGN binding (Martinez-Nunez et al., 2009), reducing the HIV infection. The over-expression and inhibition experiments with miR-155 along with TLR3 stimulation demonstrated that miR-155 inhibits HIV-1 at a step prior to the integration of the viral cDNA into the host genome (Swaminathan et al., 2012).

#### Anti-HIV miRNAs affect the expression of HDFs

Certain cellular miRNAs have been shown to inhibit viral replication indirectly through alteration of the levels of certain human proteins, termed “HIV-1 Dependency Factors” (HDFs). Inhibition HDFs has been shown to affect HIV-1 replication (Brass et al., 2008; Goff, 2008).

miR-198 is abundantly expressed in monocytes and miR-27b in resting CD4^+^T cells. miR-198 and miR-27b reduce cyclin T1 expression thereby inhibiting HIV-1 replication (Sung and Rice, 2009; Chiang et al., 2012). Cyclin T1/CDK9 heterodimer, which form positive elongation factor B (p-TEFb), is a crucial HDF for HIV-1 transcription and translation (Rice and Herrmann, 2003; Hoque et al., 2011). Following monocyte to macrophage differentiation, miR198 expression is decreased and cyclin T1 is expressed at high levels. Sung et al. provided evidence that miR-198 over-expression inhibited HIV-1 replication in macrophages (Sung and Rice, 2009). Chiang et al. later reported that miR-198 is expressed at very low levels in resting CD4^+^T cells and is not modulated upon activation (Chiang et al., 2012), supporting the concept that miRNAs exert cell-type-specific effects. Triboulet et al. showed that the miRNA cluster miR-17/92 down-regulates p200-CREB binding protein associated factor (PCAF) (Triboulet et al., 2007). PCAF is an important factor for Tat acetylation and HIV-1 LTR-driven transcriptional up-regulation (Deng et al., 2000; D'Orso and Frankel, 2009). Specifically, miR-17-5p and miR-20a over-expression resulted in PCAF inhibition, which is associated with HIV-1 transcription inhibition.

As mentioned in the previous section, miR-155 has been reported to inhibit HIV-1 infection at pre-integration step (Swaminathan et al., 2012). This inhibition is through reduction of the levels of three HDFs: (i) lens epithelial-derived growth factor (LEDGF), (ii) nuclear pore complex protein (Nup)153, and (iii) ADAM10. LEDGF, a cellular cofactor of HIV-1 integrase, promotes viral integration (Ciuffi et al., 2005); Nup153 participates in the nuclear import of the HIV-1 pre-integration complex (Woodward et al., 2009); and ADAM10 facilitate replication at the level of nuclear trafficking (Friedrich et al., 2011). Swaminathan et al. have shown that miR-155 leads to a combined inhibition of these three HDFs, resulting in accumulation of late reverse transcripts and significantly decreasing the viral integration (Swaminathan et al., 2012).

The expression of another well-characterized HDF, purine-rich element binding protein α (Pur-α) was reported to be significantly lower in monocytes than in MDDCs, which is assumed to contribute to the lower susceptibility to HIV-1 infection in monocytes (Gallia et al., 1999; Wortman et al., 2000). Certain cellular miRNAs targeting Pur-α mRNA have been identified. Those miRNAs include miR-15a, miR-15b, miR-16, miR-20a, miR-93, and miR-106b, which are highly expressed in monocytes (Shen et al., 2012). The inhibition of these miRNAs increased the Pur-α expression enhancing the HIV-1 infection in monocytes.

Table 1 summarizes the list of cellular miRNAs identified *in vitro* in relation to HIV/AIDS, the functions of which are discussed in this review.

**Table 1:** **Cellular mi-RNAs identified in HIV: *in vitro* reports**.

**microRNA**	**Target**	**Function**	**References**
miR-29a, miR-29b	Nef	Decrease HIV infection	Coiras et al., 2009; Van Lint et al., 2013
miR-149	Vpr	Decrease HIV infection	Coiras et al., 2009; Van Lint et al., 2013
miR-324	Vif	Decrease HIV infection	Coiras et al., 2009; Van Lint et al., 2013
miR-378	vpu	Decrease HIV infection	Coiras et al., 2009; Van Lint et al., 2013
miR-29c		Decrease HIV infection	Van Lint et al., 2013
miR-149, miR-147, miR-138, miR-513, miR-516, miR-518, miR-581, miR-644, miR-646	3′ UTR	Decrease HIV infection	Van Lint et al., 2013
miR-28, miR-125b, miR-150, miR-223, miR-382	3′ UTR	Latency induction in Naïve T-cells	Lecellier et al., 2005
miR-125b	Genes necessary for T-cell differentiation	Latency induction in Naïve T-cells	Hariharan et al., 2005; Lecellier et al., 2005; Nathans et al., 2009
miR-93, miR-148b, miR-221, miR-16		Decrease HIV infection	Huang et al., 2007
miR-27b, miR-29b, miR-150, miR-198, miR-223	Cyclin-T1	Restricts HIV replication	Wang et al., 2009; Rossi et al., 2011; Chaudhuri et al., 2013; Van Lint et al., 2013
miR-132	Decreases MeCP2	Increases HIV infection	Yeung et al., 2005; Chiang et al., 2012
miR-222	CD4 receptor	Repress CD4 expression in infected cells	Chiang et al., 2013
miR-146a	CXCR4	Prevents HIV entry	Desplats et al., 2013; Leoh et al., 2013
miR-217, miR-34a, miR-142	SIRT1	Increase HIV infection	Spinello et al., 2011; Orecchini et al., 2014; Quaranta et al., 2015
miR-182	NAMPT, SIRT1	Increase HIV infection	Zhang et al., 2012a
miR-155	DC-SIGN expression, DC maturation	Reduce HIV infection,	Noorbakhsh et al., 2010
	Targets HDFs (LEDGF, Nup153 and ADAM10)	Reduce viral integration	Sisk et al., 2012
miR-17/92, miR-20a	PCAF	Inhibit HIV replication	Brass et al., 2008
miR-15a, miR-15b, miR-16, miR-20a, miR-93, miR-106b	Pur-α	Low susceptibility to HIV infection in monocytes	Woodward et al., 2009

### Anti-HIV miRNAs in HIV infection in patient cohorts

Even though few *in vitro* reports have been published, only a limited number of studies have been attempted to explore miRNA profiles in HIV-1-infected patients. Houzet et al. was one of the few to report miRNA profiling in patient cohort for the first time (Houzet et al., 2008). They found that compared to uninfected healthy controls, 59 miRNAs were down regulated and 4 miRNAs up-regulated in HIV seropositive individuals. Furthermore, T-cell-specific miRNAs miR-150, miR-191, miR-223, miR-16, and miR-146b were down-regulated in all seropositive individuals, when they explored miRNA changes in specific subsets of HIV-1 susceptible cells. Among these, miR-150 and miR-223 are signature anti-HIV-1 miRNAs that have been reported to directly inhibit HIV-1 transcription (Huang et al., 2007). Huang et al. demonstrated an enhancement in HIV-1 production when all five of the “anti-HIV-1 miRNAs” (miR-28, miR-125b, miR-150, miR-223, and miR-382), they identified *in vitro* were inhibited in resting CD4^+^T cells from cART treated patients with undetectable viremia (Huang et al., 2007).

Witwer et al. described the down-regulation of miR-125b, miR-150, and miR-29 in both elite suppressors and viremic patients, compared to uninfected controls (Witwer et al., 2012). miR-155, which inhibit HIV-1 infection in macrophages (Swaminathan et al., 2012), was significantly higher only in viremic patients as compared to ES or healthy controls. miR-9, miR-34a, and miR-181 were up-regulated in viremic patients. *In vitro* data showing the up-regulation of miR-34a by the Tat, with subsequent down-regulation of SIRT1 and enhanced viral translation supports the increase in miR-34a (Zhang et al., 2012a). In a subsequent study, Witwer et al. found lower miR-125b, miR-31, miR-146b, and miR-29a expression in HIV-1-infected ART naïve patients (Witwer and Clements, 2012). Duskova et al. have noted in their study in PBMCs that chronically HIV-1-infected patients have significantly increased miR-19b, miR-146a, miR-615-3p, miR-382, miR-34a, miR-144, and miR-155 compared to uninfected healthy controls (Duskova et al., 2013).

A study by Bignami et al. found 23 differentially expressed miRNAs between exposed uninfected (EU) and healthy individuals (Bignami et al., 2012). miR-28-5p, miR-125b, and miR-223 were significantly lower in resting CD4^+^T cells from EU individuals. Eventhough many miRNAs were differentially expressed between HIV-1 infected patients and EU, Bignami et al. found only miR-155 to be significantly higher in LTNP than in naive HIV-1 patients and MEU (Bignami et al., 2012). Seddikki et al. showed a high expression of miR-155 in effector/memory Tregs compared to both naïve Tregs and naive CD4 T cells (Seddiki et al., 2013). Later on, they further found significantly lower expression of miR-9 in CD4^+^T cells from chronically infected HIV-1 patients as compared to uninfected healthy individuals or LTNP (Seddiki et al., 2013). The function of miR-9 is to bind to and inhibit the expression of B lymphocyte-induced maturation protein-1 (BLIMP-1). The BLIMP-1 expression was significantly increased in HIV-1 patients, corresponding to the lower level of miR-9, thereby uncovering a potential role for miR-9 in HIV-1-infected patients.

Reynoso et al. described that plasma miRNA profile can discriminate between elite controllers (EC) and chronic HIV infected patients (CH). They found 49 miRNAs differentially expressed between EC from CH, suggesting that higher miRNA present in EC contribute to a successful defense against HIV progression to AIDS compared to CH. The plasma expression levels of miR-29b-3p, miR-33a-5p, and miR-146a-5p were higher in EC than CH, in accordance to recent studies in PBMC. miR-29b-3p is known to target Nef (Ahluwalia et al., 2008) and an association between Nef function and slower progression to AIDS has been established (Cruz et al., 2013). No significant differences were observed between elite controllers and healthy donors; however, 16 miRNAs were different in the plasma of chronic infected vs. healthy donors (Reynoso et al., 2014). miR-18b-5p, miR-126-3p, let-7d-3p, and miR-18a-5p correlated positively and miR-424-5p and miR-34a-5p correlated negatively with CD4^+^T cell counts.

### miRNA in neuro-AIDS

A subset of individuals infected with HIV-1 develops HIV-associated neurocognitive disorders (HAND) at later stage of the disease even after successful antiretroviral therapy. It is accepted that HAND results from an indirect neurotoxicity, since HIV does not infect neurons in the brain. Activation of macrophages/microglia is a key player in the development and progression of neuro-AIDS. HIV induced miRNA dysregulation in brain targets diverse biological processes, including neuroinflammation, metabolic processes, and cell death. Micro-RNAs that have been reported in neuroAIDS are listed in Table 2.

**Table 2:** **mi-RNAs in NeuroAIDS**.

**microRNA**	**Target**	**References**
miR-21	MEF2C	Seddiki et al., 2013
miR-146a	MCP-2	Cruz et al., 2013
miR-128a	SNAP25	Yelamanchili et al., 2010; Reynoso et al., 2014
miR-125a, miR-22	IFITM3, sTNFR1A	Rom et al., 2010
miR-219	NMDA receptor	Eletto et al., 2008

Postmortem brains of HIV/SIV-infected humans and monkeys had high miR-21, miR-142-3p, and miR-142-5p (Yelamanchili et al., 2010) compared to uninfected. Myocyte enhancer factor 2C (MEF2C), a CNS transcription factor, is a target of miR-21 in neurons. Repression of MEF2C by miR-21, is a potential pathogenic factor in neurodegenerative disorders such as HAD and HAND (Yelamanchili et al., 2010). Similar to CD4 T cells (Motsch et al., 2007), HIV-1-infected primary human fetal microglia also expresses increased level of miR-146a (Rom et al., 2010) during viral infection. In cultured microglia, a negative correlation exists between miR-146a and monocyte chemotactic protein-2 (MCP-2), which is a ligand for C–C chemokine receptor type 5 (CCR5). i.e., increased expression of miR-146a leads to MCP-2 inhibition. However, they did not find any interference with viral replication with activity of miR-146a.

Six miRNAs (374, 128a, 128b, 100, 25, and 99a) were upregulated and seven miRNAs (let-7e, 298, let-7f, let-7c, let-7b, 320, and 214) were downregulated in rat primary cortical neurons exposed to Tat (Eletto et al., 2008). Tat mediated increase in miR-128a activity in neurons leads to a reduction in synaptosomal-associated protein 25 (SNAP25) expression, a key regulator of membrane fusion (Berkhout, 2008; Eletto et al., 2008). This suggests that Tat-mediated upregulation of miR-128a could lead neuronal damage.

The expression of miR-219, miR-125a, and miR-22 has been reported to be high in HIV or HIV/major depressive disorder (MDD) (Tatro et al., 2010). miR-125a and miR-22 expression in turn leads to decreased protein translation of interferon-induced transmembrane protein 3 (IFITM3), and soluble tumor necrosis factor receptor (sTNFR1A) in primary human neuronal cultures. IFITM protein is shown to inhibit HIV replication (Lu et al., 2011). TNFR1A is involved in neuroinflammation. miR-219 was shown to modulate NMDA receptor-mediated neurobehavioral dysfunction (Kocerha et al., 2009). Noorbakhsh et al. have detected altered expression of miR-129, miR-129-3, and miR-130 in HIV encephalitis (HIVE) brains. Caspase-6, -7, -8, and -9 were associated with multiple miRNAs that were suppressed in HIVE brains (Noorbakhsh et al., 2010).

#### Effect of drugs of abuse on anti-HIV miRNA expression

Intravenous drug users (IVDU) have a higher incidence of HIV in the United States and other regions in the world. Drugs of abuse are an extremely complex system, with many unknowns and/or missing links to be filled up. Drugs of abuse such as amphetamines, cocaine, marijuana, and opiates may contribute to the increased susceptibility to HIV infection and disease progression, by manipulating different genes or proteins that are needed for the HIV infection/replication. This is a well-established field, which have been published and reviewed in the past. Few examples include: (a) the upregulation of chemokine receptors (CCR5, CXCR4 etc.,), which are the co-receptors for HIV entry, (b) up-regulation of DC-SIGN in astrocytes and dendritic cells, (c) increased production of IL-10 by cocaine in macrophages which aids in HIV-1 replication, (d) Meth decreases CC chemokine expression by dendritic cells etc., (Li et al., 2002; Fiala et al., 2005; Nair et al., 2005; Reynolds et al., 2006; Dhillon et al., 2007; Liang et al., 2008; Shapshak et al., 2011). In addition to the co-receptor or entry receptor modulation, *in vitro* and animal model experiments have also shown that drug use affect immunologic components that, in turn, influence HIV disease progression. Cocaine has shown to disrupt the immune functioning (reviewed by Baldwin et al., 1998; Hauser and Knapp, 2014; Pandhare et al., 2014; Parikh et al., 2014). These drugs have also been reported to alter the expression of some anti-HIV miRNAs, which are listed in Table 3.

**Table 3:** **Drugs of abuse and anti-HIV miRNAs**.

**Drugs**	**Effect on mi-RNA expression**	**References**
Morphine	Decrease miR-28, miR-125b, miR-150, miR-382	Wang et al., 2011
	Increase miR-181b	Dave and Khalili, 2010
	Decrease miR-15b	
	Decrease miR-155 and miR-20a	Dhillon et al., 2007
Cocaine	Decrease miR-155 and miR-20a	Fiala et al., 2005
	Decrease miR-146a	Kocerha et al., 2009
	Decrease miR-125b	Li et al., 2002
Methamphetamine	Increase miR-28, miR-125b, miR-150, miR-223	Shapshak et al., 2011
	No change in miR-296	

Morphine exposure has been shown to change the expression of cellular anti-HIV miRNAs in monocytes *in vitro*. The expression of four IFNα/β inducible anti-HIV miRNAs (miRNA-28, miRNA-125b, miRNA-150, and miRNA-382) were decreased in monocytes treated *in vitro* with morphine, compared to untreated cells (Wang et al., 2011). These miRNAs were correlated with the susceptibility of monocytes to HIV-1 infection, demonstrating a plausible mechanism for morphine-mediated enhancement of HIV infection of monocytes. Same miRNAs were lower in PBMCs from uninfected, heroin abusing individuals compared to healthy, non-abusing individuals, corresponding to *in vitro* findings. Another anti-HIV miRNA, miR-223, was not affected by morphine, the reason for which is not clear. *In vivo* investigations using PBMCs from the heroin-dependent subjects also showed the same results for miR-223 expression.

Dave and Khalili reported 26 differentially expressed miRNA in human MDMs treated with morphine; with miR-15b expression showing greatest increase and miR-181b greatest decrease (Dave and Khalili, 2010). Morphine induces inflammation and oxidative stress in immune cells through regulating the miR-15b and 181b, thereby contributing to the AIDS progression.

Drugs of abuse have shown to upregulate the expression of CXCR4, which facilitate the entry of HIV into CD+4 T cells resulting in increased infection of X4-tropic HIV-1 (Steele et al., 2003). We, in our preliminary *in vitro* experiments saw that cocaine down-regulated miR146a thereby increasing CXCR4 expression (Pilakka-Kanthikeel et al., 2012). We have also shown that cocaine and morphine significantly down-regulated miR-155 and miR-20a in MDDC, thereby enhancing the HIV-1 infectivity. Cocaine or morphine induced effect on HIV infectivity was reversed by transfection of MDCC with miR155 mimic (Napuri et al., 2012, 2013).

miR-125b is a member of anti-HIV-1 miRNA family that targets the 3′-UTR of HIV-1 transcripts and inhibit viral translation, a post entry step (Rossi et al., 2011). Cocaine, was also shown to inhibit miR-125b in CD4^+^T cells, which in turn enhances HIV-1 replication (Mantri et al., 2012). The over-expression of miR-125b decreases HIV-1 replication, suggesting a key role for miR-125b in the cocaine-induced enhancement of HIV-1 replication in CD4^+^T cells.

METH has been shown to increase HIV-1 replication in various HIV-1 permissive cells, including dendritic cells (DCs) (Nair et al., 2009) and monocyte-derived macrophage (Liang et al., 2008). Toussi et al. showed enhancement of HIV-1 replication of R5 tropic JR-CSF HIV-1 in human CD4^+^T cells and in the peripheral CD4^+^T cells of JR-CSF/hu-CycT1 HIV-1 transgenic mouse, with METH treatment upto 150 mmol/L (Toussi et al., 2009). In contrast to these reports, a recent study by Mantri et al. showed that METH inhibits HIV-1 replication by up-regulating the cellular anti-HIV-1 miRNAs (miR-28-5p, miR-125b, miR-150, and miR-223) in primary CD4^+^T cells (Mantri et al., 2014). The expression of the anti-viral miR-296-5p was not affected by METH in primary CD4^+^T cells.

## Conclusion and future directions

Taken together, the present findings suggest that cell type specific expression of intracellular anti-HIV miRNAs play a role in making cells more or less susceptible to HIV infection. miRNAs have been suggested to play role in maintaining the HIV latency, the transition from latency to activation, the reduction of virion production etc. However, the interaction of the host miRNA with HIV-1 is still at a budding stage. A complete cure for HIV-1 is possible only when HIV-1 gene is silenced or completely eliminated from every latently infected cell. Identifying the role of miRNAs associated with HIV-1 latency could help in developing new strategies to intervene the mechanism of viral persistence. Also, more studies on the impact of drugs of abuse on anti-HIV miRNA expression, and strategies to block their effect on miRNA expression will help in developing therapeutics for drug addiction by manipulating the actions of miRNA.

An interesting avenue for future research that has not yet been explored is to investigate if HIV-1 modulates the expression of cellular miRNAs that can alter the expression of restriction factors. Interestingly, miR-181 (miR-181- a,b,c and -d) has been predicted to bind to and potentially inhibit the HIV-1 restriction factor sterile alpha motif and histidine/aspartic acid domain-containing protein 1 (SAMHD1). We and another group recently shown miR181a and miR-155 regulate SAMHD1 (Jin et al., 2014; Pilakka-Kanthikeel et al., 2015a,b). Earlier, Gottwein et al. reported a viral miR-K12-11, an ortholog of cellular miR-155, to target SAMHD1 (Gottwein et al., 2007). More studies are warranted to study the role of miRNAs that regulate the HIV restriction factors and how drugs of abuse interact with them.

### Conflict of interest statement

The authors declare that the research was conducted in the absence of any commercial or financial relationships that could be construed as a potential conflict of interest.
